# A conceptual framework for psychosocial health management grounded in the therapeutic merits of indigenous KhoiSan health dialogues

**DOI:** 10.4102/hsag.v26i0.1626

**Published:** 2021-08-16

**Authors:** Khauhelo S. Mahlatsi, Abel J. Pienaar, Neo E. Nare, Tshilidzi M. Mulaudzi

**Affiliations:** 1 Department of Nursing, Faculty of Health Sciences, University of Venda, Thohoyandou, South Africa; 2 Department of Psychology, Faculty of Health Sciences, University of Venda, Thohoyandou, South Africa; 3 Department of Graduate Studies and Research, Faculty of Nursing and Midwifery, Shifa Tameer-e-Millat University, Islamabad, Pakistan; 4 Department of Health, Mental Health, North West Provincial Government, Mmabatho, South Africa

**Keywords:** communal counsellee, communal counsellor, communal individual, dialogues of healing, healing, psychosocial health, *!nxabasas*, *mpho ya badimo*, the wheel of life

## Abstract

**Background:**

The researchers established that an indigenous KhoiSan community functions healthily without western mental health services. This community relies on indigenous healthcare with positive health outcomes over centuries. Despite this positive evidence, the community’s therapeutic achievements have not been explored previously.

**Aim:**

To explore the therapeutic merits embedded in dialogues of healing to formulate a generic approach to managing psychosocial challenges.

**Setting:**

The study was conducted in an indigenous KhoiSan community, Northern Cape province, South Africa.

**Methods:**

A qualitative approach, by using an indigenous African research design, was followed. An African Indigenous Health Research Framework (AIHRF) was employed, particularly applying a classical African indigenous method of data-collection, namely orature. Theoretical sampling was used for the purpose that the emerging data guide the researcher to the next participants. The four-step analysis of the mentioned framework was deployed for data analysis.

**Results:**

It was deduced that the therapeutic merits of dialogues go beyond the word of mouth, leading to the emergence of themes related to the successful management of psychosocial health challenges in the KhoiSan community.

**Conclusion:**

These findings were used to generate a baseline conceptual framework for the management of psychosocial challenges in the KhoiSan community.

**Contribution:**

Revitalisation of communal indigenous practices for the management of psychosocial health challenges within the KhoiSan community. The latter will sensitise research, teaching and learning to foster culturally informed counseling approaches. Moreover, these will inform policy formations to posses a culturally competent approach towards indigenous communities such as the KhoiSan community in the Northern Cape, South Africa.

## Introduction and background

‘Unabatedly Indigenous Health Systems in Africa stood the test of time’. This statement conveys the resilience of African indigenous healthcare, despite the evolution and operationalisation of western medical systems in Africa.

Consistent with the above understanding, Moshabela, Zuma and Gaede (2016) assert that indigenous health practices in South Africa remain functional in indigenous communities, although unrecognised. Following this lack of recognition and support by means of policies, Africans have had to adopt western healthcare systems. However, Salamonsen and Ahlzén (2018), Malokoane et al. (2020) and Rispel (2016) argue that a wide range of states across the world have adopted and implemented western health systems with ineffective outcomes and increasing levels of patient distrust. These authors further contend that many western health systems in Africa are under-funded, under-researched and under-resourced, leading these mechanisms into struggling to cope with the cumulative burden of diseases (Malokoane et al. 2020).

Furthermore, Burger and Christian (2020) find that South African history has exposed the demeaning effects on the health of its people in relation to the western health policies and current health services. These policies are apparent in the promulgation of suppressive laws such in Section 1 of the *Witchcraft Suppression Act 3 of 1957*, which, unfortunately, has not been repealed even in the current democratic dispensation. Adding to the latter, The South African Pagan Rites Alliance (SAPRA) alleges: ‘South African law currently presumes that the practice of “witchcraft” may be used to commit or justify the commission of criminal activity’ (SAPRA, 2012). Sadly, we cannot move past the annals of history without observing the destructive impact of colonisation on the indigenous African health systems. Therefore, the researcher envisioned in revitalising and re-inscribing the authentic practices of Africans by illuminating the benefits of their indigenous health system, specifically dialogues of healing.

### Statement of the research problem

Clearly, from the previous discussion, African indigenous healthcare has withstood the test of time. Despite the suppression by colonialist laws in South Africa, the indigenous healthcare system remains resilient in communities like the KhoiSan community in the Northern Cape. In the researcher’s experiential learning in this KhoiSan community, as a registered mental health nurse, it is eminent that this community survives without a psychologist, social worker or mental health nurse. The community copes with their own psychosocial health practices, building resilience. Although the resilience of this African indigenous healing approach is distinctly distinguished, the exploration of its therapeutic merits has not been researched and explored. Therefore, the researcher strove to explore the therapeutic merits, linked to dialogues of healing that this community utilises in managing their psychosocial challenges.

### Aim and objectives

The aim was to explore the therapeutic merits embedded in dialogues of healing and subsequently developing a generic approach to managing psychosocial challenges in a KhoiSan community in the Northern Cape. In line with this aim, the following objectives were designed to:

- explore the dialogues of healing
- elucidate the benefits of these dialogues in relation to psychosocial healing
- revitalise and re-inscribe the healing practices embedded in ‘dialogues of healing’ for the management of psychosocial challenges.

### Conceptual definitions

**Communal individual** refers to a person(s) who is seen as a whole (body, mind, emotions and spirit), connected to other persons, surroundings, and those who were here before us, living on the principle of sharing and caring.

**Communal counsellor** refers to a person (especially an elder) who offers guidance, healing and teaching to the communal individual in need of support.

**Communal counsellee** refers to a person who receives guidance, healing and teaching from the communal counsellor.

**Healing** is a sacred process grounded on epistemologies and praxis, which seek for a balance in the connectedness of a person as whole (mind, body, spirit, the environment and those who were here before us – ancestry).

**Dialogues of healing** is a therapeutic and shared process, where the ownership remains with the sharer, although the resolution is carved collectively by the indigenous people involved. It is performed with a strong belief in the wholeness and spiritual connectedness to the cosmos whilst they mediate to achieve an equilibrium in life.

The researcher advances **psychosocial health** as a complex phenomenon which views a human being as a collective attached to a communal heritage with its own interpretation of the present and the past.

**Those who were here before us.** This is defined as a person (body, mind and spirit) who has passed on and is appreciated as being part of the physical surroundings, in line with the belief that this person has passed to the next life with a different role of guiding and guarding the broader community.

**Those who passed on.** This expression is used interchangeably with ‘those who were here before us’.

**!nxabasas**. This is a special practice used to purify young adolescent girls.

## Research methodology

### Research design and framework

A qualitative approach, following an African indigenous research design was employed, guided by the African Indigenous Health Research Framework (AIHRF). This framework proposes that ‘research needs to be grounded in its authentic research context’ (Pienaar 2017).

The AIHRF deals holistically with psychological and social issues (Pienaar 2017). It is used in the joint context of psychology and sociology with the purpose of keeping indigenous meanings intact (Pienaar 2017). The research was a negotiated partnership, allowing the indigenous KhoiSan community to define the extent to which they saw fit to make themselves available as research participants. The chief of the KhoiSan community was engaged to consent to the study. The matter was first informally and confidentially brought to the chief’s attention (Pienaar 2017).

The researcher followed a classical (conventional) African indigenous approach. Pienaar (2017) proposes that the researcher grounds the study on an African worldview, where the framework, without dilution, is based on African indigenous knowledge systems.

### Study site

The research was carried out in an indigenous KhoiSan community in the Northern Cape province of South Africa.

### Population

The research population is an African indigenous community, namely KhoiSan, domiciled in the Northern Cape province of South Africa. This is a small town of not more than 5000 inhabitants, situated in the east of Griqua-Town.

### Sampling methods

A theoretical sampling in this study was applied as traditionally used in grounded theory because of its flexibility, leading the researcher to the next participants (Butler, Copnell & Hall 2018). Furthermore, as attested by Brink, Vander Walt and Van Rensburg (2014) grounded theory is founded on the comparison of existing data with those currently collected. Thus, the sampling method was used primarily for theory generation and for its ability to guide in emerging theory (Glaser & Strauss 1999). Theoretical sampling was employed as discussed by Glaser and Strauss (1999) as the process of data generation for engendering theory through which the researcher jointly collected, coded and analysed data. Consequently, the researcher decides what data to collect next as led by the categories and themes. Another purpose for this sampling was to allow the emerging data to lead the researcher to the next participant without a preconceived criterion.

### Data collection methods

In this research, orature, which includes storytelling, metaphors and praise poems, was used for data collection. Reverentially, it is argued that sensitivity to African realities must be exhibited by researchers (Naidu & Prose 2018; Owusu-Ansah & Mji 2013). Hence, orature was a data collection method applicable to the culture, justifying why it was used.

After a negotiated partnership was established, the chief of the community allocated two indigenous mediators to assist the researcher in the process of data collection. Their roles and functions were to facilitate the cultural competence of the researcher, translate the spoken word from the researcher (English) to the participants (Afrikaans), and negotiate appointments with the following participants depending on the emerging conceptual framework. Moreover, the two mediators were integrally involved in the data analysis as it ran concurrently with collection. This collaborative process went on until data saturation was reached. A total of 10 orature sessions ranging from 60 to 120 min were conducted.

Data collection was carried out by asking the following four main questions: (1) ‘How do you use dialogues or conversations to heal psychosocial challenges?’ (2) ‘Do you have any stories within the community that could assist in the healing of psychosocial challenges?’ (3) ‘Do you have any praise poems that could assist in the healing of psychosocial challenges?’ (4) ‘Are there any metaphors (sayings) in the community that assist with psychosocial healing?’

### Trustworthiness of the research

Lincoln and Guba (as quoted in De Vos et al. 2009; Liamputtong 2009) was followed for the trustworthiness of the results.

Firstly, credibility was achieved by crystallisation where the researcher collected data from different participants depending on the emergence of theory. Secondly, for member checking, the researcher returned to the participants to confirm the original findings. Lastly, data collection methods by nature integrally have the participants as part of the research. Confirmability, which refers to the objectivity of the data through an internal agreement, was achieved by returning to the community and verifying the raw findings.

### Ethical considerations

Firstly, approval was obtained from the North-West University (NWU) Ethics Committee (NWU-00724-17-A9). The written memorandum of agreement and understanding (MOU) were reached with the KhoiSan King and the community. The MOU and the research adhered to five general principles provided by the American Psychology Association (2017). Overall, the MOU gives guidance on benefit sharing between the community and the researchers. For that reason, this conceptualised practice remains the property of the community. Hence, the dignity of the participants and self-determination was respected. Thus, based on approval by the Health Science Ethics Committee (FAST-HSEC) on 03/03/2018, the North-West University Research Ethics Regulatory Committee (NWU-RERC) hereby approved this research. This implied that the NWU-RERC granted that the project may be initiated.

### Data analysis

The researcher recorded and kept the audio recordings for data processing and transcriptions. The audio recordings were collated daily for translation from Afrikaans to English by the two indigenous mediators, and then these were used for reflective purposes, in addition to the notes recorded during data collection.

A four-step analysis according to the AIHRF was used (Pienaar 2017) as follows.

*Level one:* ‘Basic concepts from the spoken word’. The researcher and participants collected and analysed data instantaneously.

*Level two:* ‘Joining or grouping of similar concepts to form a cluster’. Constantly, as the concepts formed, the researcher in collaboration with the participants separated and conjoined related concepts.

*Level three:* The researcher, in collaboration with the participants, intuitively deduced, converged and identified new concepts, themes or clusters (insights and/or discoveries).

*Level four:* This is the building of a story line or pattern to form a process and a generic conceptual framework for the African context.

## Discussion of findings

Initially, it is important to note that *levels one* and *two* of analysis occurred during data-collection to inform the subsequent levels. During *level three* of the analysis, the researchers, in collaboration with the participants, intuitively deduced new concepts and themes (Pienaar 2017).

Following the findings came out of the analysed data, meaning is further crystallised through sub-theming and categories under the umbrella themes – indigenous communication systems and communal lifestyles (see Table 1). These are two rudimentary themes that were integral to the understanding of the merits of dialogues through ‘who’, ‘why’, ‘how’, ‘when’, ‘what’ and ‘whom’.

**TABLE 1:** Main themes, sub-themes and categories.

Theme	Sub-theme	Categories
1. Indigenous communication systems	1.1 Spoken word	Silence Metaphors (reprimanding and/or educational) Stories
	1.2 Active communication	Self-resilience disclosure Directive and instructive language
	1.3 Principles and attitudes of communication	Humility; softness; Don’t be clever or strong; Silent communication; Covert speech; Gender-based societal challenges (conversation for men and conversation for women); Process of readiness and non-readiness (reprimand); Patience and/or respectful waiting; Boundary setting; Direct and Instructive language; Self-disclosure (resilience)
2. Communal lifestyle	2.1 Day-to-day practices	Behavioural conduct Eating habits
	2.2 Special practices	Rite of passage -*!nxhabasas* Grave-yard visit Commemoration *Mpho ya badimo*

*Source*: Mahlatsi, K.S., 2018, *Dialogues of healing in the management of psychosocial health challenges in an indigenous KhoiSan community,* Masters thesis, School of Nursing Sciences, North-West University

### Theme 1: Indigenous communication systems

This theme was emerged when the participants repeatedly emphasised the importance of ‘*who* is saying *what* to *whom*; *when* and *why* and *how* they are saying it’ to the communal person(s), family or community. Explicitly, how one is communicating to another is a pivotal element in the facilitation of healing in one’s dialogue.

Indigenous communication systems play a key role in achieving balance of life through dialogue. It is worth mentioning that context, people and time matters. This means the communication system of one context can be incomprehensible to another social context. This view of this communication system is shared by Abdulai, Ibrahim and Mohammed (2017) who argue that the interpretation of what is being communicated may vary from one society to the other.

From the theme indigenous communication systems, three sub-themes emerged: *spoken word*; *active communication*; and *principles and attitudes during communication*.

In sub-theme (1.1), *spoken word* is a word of mouth that is deeper than what is said on the surface. As complex as it sounds, it became clear that the community listens not only with the ears, but with all the senses: eyes, touch and smell for the deeper meanings. Timeously, it emerged that what was being said could also reflect someone’s past or future. Hence, there was an immediate realisation that the meaning of what was being said could lie with those who have passed on.

Seamlessly, with category (a), *silence* within the KhoiSan community represents the meaning of what is not being said (listening between the verbal lines). It emerged that words and silence are powerful and integral to the communication practices of the KhoiSan. Ouzman (2005) and Blanco (2018) articulate that the communicative load of a word relies on the quality and texture of the silence. Likewise, the KhoiSan community’s words and silence hunt together where an appreciation is shown to their holistic meaning. Thus, it became clear that words and silence are powerful forms integral to the structure of a specific communication system:

‘We know when to say nothing and still deliver deep-rooted message.’ (Participant B1, female, 68 years old)

Consequently, with category (b), *metaphors*, a realisation came that they are often used by elders for educational reasons and when reprimand is needed:

‘If you plant a pumpkin, you cannot expect a peach.’ (Participant N5, male, 60 years old)

‘You cannot plant thorns and expect apricots.’ (Participant J2, female, 60 years old)

‘Early ripe, early rotten.’ (Participant A1, female, 69 years old)

With these metaphoric paradoxes, the communal individual’s future does not seem bright if she or he continues with the misconduct. As expressed, these metaphors were normally used with reference to girls who move into womanhood at a young age. The intended teaching is to caution the youth against early sexual engagement unless they want to lose their integrity. These metaphors are used for either education or reprimand:

‘A rude child’s dumpling doesn’t cook well.’ (Communal healer, female, 58 years old)

‘A rude child’s lamp dies in the darkness.’ (Participant C2, female, 88 years old)

These statements mean children should respect adults. Also, those portraying bad manners mostly are likely not to succeed in life. Clearly, these metaphors are significant resources for educational purposes and reprimand used by elders to guide and nurture the youth.

Lastly, in the category (c), ***stories***, it emerged that resilience is at the centre of the stories told. Likewise, indigenous ways of living have withstood the test of time despite the translocation and evolution of western civilisation. These stories are either told in remembrance of those who were here before us or a reflection of their day-to-day life:

‘We had to travel far to get water.’ (Participant D2, male, 72 years old)

‘We had to work long hours for the whites, paying us cents.’ (Participant B1, female, 68 years old)

Meanwhile, it emerged from the stories that resilience is important in managing psychosocial challenges and social cohesion. Similarly, the story in Box 1 is that of a ‘phokoje’ (jackal) on social cohesion. The ‘jackal’ in this nugget refers to the ‘men preying on young girls’ where young girls are metaphorically referred to as sheep.

BOX 1 The story of *phokoje* [jackal].

This story was narrated by an elderly woman (Participant E1, female, 100 years old) where she focussed on a psychosocial communal practice of protecting younger girls from men with predatory behaviour. On a playground, a group of young girls (sheep) and a young boy disguised as a jackal appearing to be preying on the sheep (young girls). Wherein, another young boy (a hero) appears to be a protector who by all means would chase the jackal away from the young girls.

*Source:* Adapted from Mahlatsi, K.S., 2018, *Dialogues of healing in the management of psychosocial health challenges in an indigenous KhoiSan community*, Masters thesis, School of Nursing Sciences, North-West University

Essentially, the above story demonstrated the sense of protection and responsibility amongst the youth in the KhoiSan community. It was found that the younger girls felt protected, cared for and loved. A sense of communality and togetherness amongst the youth is embedded in this nugget. Consequently, it emerged from sub-theme 1 and its categories that the notion of ‘who’, ‘whom’, ‘how’ and ‘what’ is of significance in the appreciation and understanding of the context. In simple terms this means, ‘what’ and ‘how’ as a communal counsellor one speaks to the communal counsellee and ‘what’ you want to achieve. To demonstrate, it emerged that the above story represents the ‘what’, whereas the ‘how’ represents the metaphoric form in which the story is told. In effect, the ‘who’ represents the elderly woman who told the story through experience and the ‘whom’ represent the youth who have to be educated, thereby bringing about the virtue of togetherness.

With sub-theme (1.2), active communication, the realisation occurs that there is also the ‘action part’, which complements the spoken word. In illustration, action brings more decisiveness in the usage of indigenous communication systems. By inference, this action is realised in the form of *direct and instructive language* which is always coupled with a reprimand. It is, however, characterised by a touch of gentleness through *self-resilience disclosure* where a communal counsellor opens up about their own experience. At the same time, this is done recursively without anger until healing is achieved. Holistically, this is found to be where the communal counsellor opens to the communal individual at the centre of the psychosocial challenge. It is when the counsellor(s) narrate their own lived experiences. As a result, there is a communal rapport. This technique also shows empathy and guidance on how to improve as the counsellee:

‘A problem is a problem, and no problem is bigger than a family.’ (Participant D2, male, 72 years old)

‘I have been through this I know how you are feeling.’ (Participant E1, female, 100 years old)

‘Life might seem like it’s unfair to only you, but we all have been through this.’ (Participant C2, female, 88 years old)

There is a connection that became clear in sub-theme 1.2 and its categories inherently address the ‘what’ and ‘how’ to say to the communal counsellee when managing psychosocial challenges by means of dialogues.

Sub-theme 1.3, *principles and attitudes of communication:* This sub-theme emerged when participants repeated the importance of the attitude and makeup of the communal counsellor. The importance of the healthy and conducive atmosphere (yourself as the helper and the environment) should always be welcoming and conducive for healing. This sub-theme and its categories inherently attend to the ‘how’, ‘what’, ‘who’ and ‘whom’ as the communal counsellor should discharge their function:

‘If the psychosocial issue involves a married couple, only elders and married individuals are allowed to manage the challenge.’ (Participant F1, female, 50 years old)

‘If the problem involves the girl child, the mother, aunts and sometimes the women of the community depending on the extent of the problem.’ (Participant F1, female, 50 years old)

‘Equally, if the problem that needs to be managed involves a boy child, only men will manage the problem.’ (Participant H1, male, 70 years old)

The communal counsellor should be *humble* and *respectful* throughout the process of psychosocial management. A communal counsellor should not carry themselves as the ‘better one’ to prove others are wrong. Everyone should be equal, not perfect. Clearly, *respectful waiting* is an important trait in the character of the counsellor. Equally important is that not everyone is involved in psychosocial management. This chore and responsibility depend on the nature of the challenge, with cognisance of gender matching. Certain challenges are managed by women, mothers and elderly women of the community, and there are those managed by married women only. This is all inextricably woven into the fabric of the ‘what’, ‘who’ and ‘whom’ question:

‘Be soft, talk with a sense of gentleness.’ (Participant M1, female, 79 years old)

‘Do not be clever or strong.’ (Participant B1, female, 68 years old)

‘Do not rush him, let him go through the change process from within.’ (Participant B1, female, 68 years old)

‘Even though I am firm and saying all this, the person still has a special place in my heart.’ (Participant M1, female, 79 years old)

‘Respect one another regardless of age.’ (Participant H1, male, 70 years old)

‘If you want to be respected, give out respect.’ (Participant C2, female, 88 years old)

In the penultimate it emerged that the communal counsellee should maintain *gentleness* whilst at the same time being firm in *setting boundaries*. Mainly, the communal counsellor assesses the *readiness for change* from the counsellee by respectfully waiting.

### Theme 2: Communal lifestyle

Interestingly, it emerged that the merits of dialogue are not only bound to the traditional linear model of communication (word of mouth ‘message’ between sender and receiver), but these are also embedded in the practices and rituals – ‘what’ of the community during different stages of life – ‘who’. With that understanding, the importance of practice and rituals emerged when participants repeatedly conversed the significance of communicating with ‘those who have been here before us’ to bring about healing and therefore maintain the balance of life.

Centrally, the importance of practice and rituals is in line with Bojuwoye and Moletsane-Kekae (2018), who state that the environment is continuously changing, causing imbalances. Thus, for survival, the African communities restore balance by regularly performing connecting rituals. It was deduced that to achieve healing, everyone must establish and maintain a form of balance with their environment. It emerged that the KhoiSan community maintains this balance with the ever-changing surroundings through the *day-to-day indigenous practices* and *special rituals*.

The emergence of sub-theme (2.1): day-to-day practices, it came out after the participants emphasised that their life is ‘one’ where they live in the strong belief that they are connected to the environment; those who passed on; and those who are still in the physical form with them. Hence, the realisation came that the way of life of this community cannot be compartmentalised. Hence, the merits of dialogues are visible in their lifestyle:

‘Those who passed on form part of us.’ (Participants B1, female, 68 years old)

‘Yes my son has passed on, I talk to my son every day, and I know he can hear me, and I also can feel and hear him.’ (Participant N5, male, 60 years old)

‘My father who passed on visited me at night while I was sleeping and touched me while I covered myself with my blankets. I asked him not to scare me, if there’s something he wants to say, he must please say and not scare me.’ (Participant F1, female, 50 years old)

The above quotes confirm and validate Du Toit (1980), who observed that there were life cycle rituals that were significant for strengthening the individual and protecting them for the new life phase.

Furthermore, with category (a) there was evidence that the communal essence of behavioural conduct towards communal lifestyle is demonstrated by these day-to-day practices which are instilled from childhood throughout the stages of life. In turn, these practices help with behavioural conduct amongst the people living in the community. Inherently, this category on behavioural conduct addresses the notions of ‘*how*’ and ‘*whom,*’ directing the way in which the people (children, youth, adults, women, men and elders) are expected to carry themselves:

‘These are our cultural practices, meant also for us to maintain the balance of life.’ (Participant D2, male, 72 years old)

‘A girl child is not allowed to sleep out.’ (Participant C2, female, 88 years old)

‘A boy child is raised to respect women.’ (Participant H1, male, 70 years old)

Equally, with the category (b), eating habits, the merits of dialogues were realised. It emerged that there are eating practices within the community, which are acceptable and specified according to the age and gender of the communal individual. This understanding emerged as representing the notion of ‘what’ and ‘who’:

‘Brain and the skull [*of a slaughtered beast*] always should be given to elderly people in the family and community.’ (79 years old, female, participant M1)

‘The backbone and its meat [*of the animal*] are meant for elderly people to eat and never children.’ (Participant E2, female, 62 years old)

‘Bones and meat of the lower parts of the front legs are for the children.’ (Participant M1, female, 79 years old)

Indigenously, the practice of giving the brain and the skull of the slaughtered animal to the elderly comes with the belief that these parts carry the knowledge, strength and protection transmitted through by those who were here before. The question on why bones and meat of the lower forelegs are offered to children represents a strong encouragement to children to aspire for higher horizons and marching for the better life.

In the same way, it became clear that therapeutic merits embedded in dialogue go beyond word of mouth in this community. These are integral to the communication system and lifestyle of the KhoiSan community. Overall, with sub-theme 2.2. this realisation showed that special practices take place at different stages of a communal individual’s life. Du Toit (1980) and Ouzman (2005) concur that in striving to maintain balance life cycle, rituals form part of that effort, and these can be performed by the household head. In some instances, an indigenous practitioner is called for guidance. In support, Edwards et al. (2009) submit that if such rituals are not performed, there is a strong belief that this would bring about imbalances resulting in sickness and bad luck as a consequence of the unhappiness of those who were here before. Similarly, Bojuwoye and Moletsane-Kekae (2018) observed that there are life-cycle rituals, which are significant for strengthening the individual and protecting them for the new life-phase in order to regain collectivity with oneself and the community.

Interwoven in the communal lifestyle, with category (a): rite of passage, this is a ritual performed to transition a communal individual to another stage of life. This was realised by participants saying in case of a menstruating young girl starting to show a behaviour of sexual activities; these young girls are taken through a ceremony referred to as ‘!nxabasas’ (purity of a young girl):

‘The ritual is for the girl to be introduced to the people who were here before us as a woman, for them to protect her and guide her.’ (Participant C2, female, 88 years old)

‘This is about teaching the girl child as woman of culture how to do things and behave herself.’ (Participant C2, female, 88 years old)

With the emergence of category (b), grave-yard visit, the essence of this visit came about when the participants insisted on the importance of visiting the graveyard. They communicated the strong belief that visiting the fore-parents’ graves bring about blessing and the balance in life:

‘If I have problems I just visit my dad and talk to him. I know he is listening.’ (Participant F1, female, 50 years old)

‘When I come back from the graveyard I know and strongly believe that my problems will go away.’ (Participant D2, male, 72 years old)

Meanwhile, the emergence of category (d), commemoration of the late King Adam Kok (IV), generates its meaning in the annual ceremony where the community especially thanks and exhibits gratitude to one of the kings of the KhoiSan community. He was born in October and died in October, the same month for birth and death, explaining why the event is always held in October of every year:

‘If I feel I have bad luck, things are not going well for me, I perform *Mpho ya badimo*.’ (Participant H1, male, 70 years old)

‘The year I found a job I did perform *mpho ya badimo*.’ (Participant E2, female, 62 years old)

Equally important, category (e) emerged – ‘*Mpho ya badimo*’, which is a Sotho derivative meaning giving back to those who were here before us. This is done when a communal individual shows respect and appreciation to those who were here before us. Through this ritual, a sacrifice is made, normally of a sheep or a goat, and it is important to maintain discipline when one is eating any part of the slaughtered animal.

**FIGURE 1:**
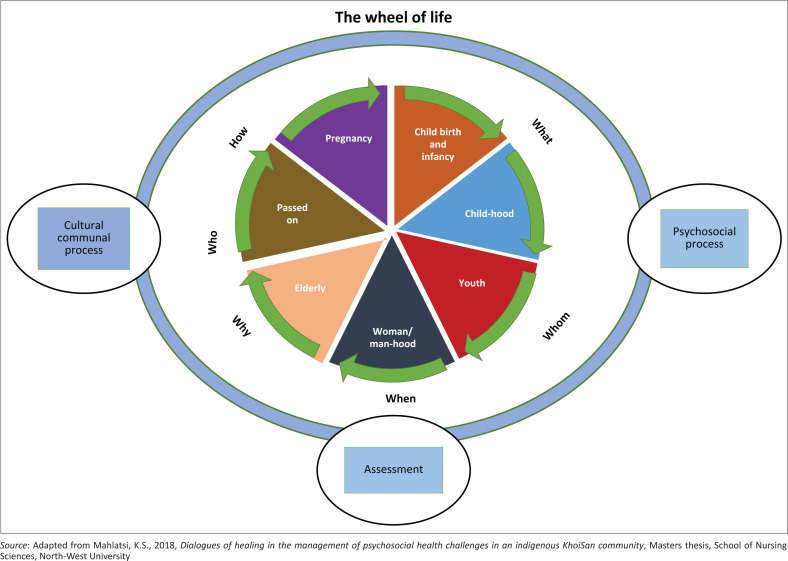
The wheel for the management of psychosocial health challenges in an indigenous KhoiSan community.

Noticeably, in exploring dialogues of healing (objective one) and elucidating its benefits (objective two), it emerged that the KhoiSan community fundamentally upholds communality in their way of life. Integrally, it was found that the therapeutic merits of dialogues have a strong meaning in ‘who’ is saying ‘what’ to ‘whom’, ‘when’ as well as ‘why’ and ‘how’ they are saying it to achieve the balance of life for healing. Dialogues of healing do not just involve the living, but also bring in those who passed on, therefore having meaning beyond what is being said and also ‘what’ is being practised as a lifestyle within the community. This discussion of the findings linked *level three* of analysis. Thus, *level 4 analysis* is explored below by building a story line or pattern to form a framework for Africans. Notably, Glaser and Strauss (1999) assert that the development of an approach is best when it emerges from the categories and core concepts in the findings. Henceforth, a conceptual framework emerged that depicts the Wheel for the management of psychosocial challenges in an indigenous KhoiSan community (see Figure 1), embedded in the life stages, African indigenous communication and rituals prescribing a communal lifestyle.

Foundationally, life stages are situated in ‘the wheel’ (Figure 1), which is underpinned by the philosophy that persons are seen as a whole, connected to the surroundings and their ancestry. For indigenous psychosocial life to be wheeled, it is dependent on the communication to the surroundings and our ancestry. It emerged that in every life stage, the communal person should be introduced through a communal ritual. Added to this, the communal person performs day-to-day practices to maintain the balance of life. Therefore, when a life is conceived (pregnancy), there are rituals performed to introduce the new life to the fore parents. This introduction personifies recognition, calling for support and protection. To keep the meaning of this new life wheeled, in the childhood stage, there are day-to-day rituals performed, mostly by the head of the family.

In transitioning from the youth stage into womanhood or manhood, a rite-of-passage is performed. The boys are taught how to behave as a responsible man; how to treat women; what is expected in a man, mostly building and maintaining a home for his family.

On the other hand, women are taught how to carry herself and how to behave as a woman and mother. They are taught dancing, cooking, respect and what is expected from a KhoiSan woman. These rituals are designed for encouragement and the promotion of respect, ultimately keeping life balanced and progressively wheeled.

Moving from womanhood or manhood into the realm of the elders is seen by being assigned more responsibility which often happens spontaneously through, for example, conducting rituals and heading family meetings. Later, when you pass on to another realm, it is believed that you are still with us and have assumed the responsibility of providing vision, caring and protecting communal persons, family and the community on the other side of life.

Living in the KhoiSan community cannot be compartmentalised but understood as ‘whole’ with the prescripts of its philosophy, ontology and epistemologies. Living is therefore viewed as a continuous journey where every age group and practices are landmarks on this journey.

It emerged that inter-communal teams are formed, given a psychosocial challenge, either at a family or community level, and are exclusively guided by the ‘wheel of life’. Hence, the approach is guided by its inseparable techniques as depicted in the diagram (Figure 1).

### Approach

An approach for the successful management of psychosocial health challenges in an indigenous KhoiSan community is interwoven with assessment, communal cultural process, and a psychosocial process. The approach is distilled below.

#### Assessment

The communal team with the counsellee makes a determination on the root of the psychosocial challenge, based on the *wheel of life*.

As previously alluded, life is ever-changing, and this brings about imbalance to the ‘wheel of life’, which translates into ill-health evidenced by a psychosocial challenge. After assessment, the counsellor critically identifies the stage of the communal counsellee. Determination would then be made on ‘why’ there is an imbalance of life. Imbalance is mainly the manifestation of the ‘unhappiness’ shown by those who were here before us. Because of the connectedness mentioned before, this unfolds in unfortunate events as the connection has been disrupted. These can be presented by a job loss, delinquent behaviour, emotional stress, mental problems and/or death in the family.

At the same time, an assessment of readiness is made whether the communal individual is ready for change. If not, the proceedings are terminated (‘when’). The counsellee may come back later; this represents characteristics of readiness for the process to re-commence.

#### Communal cultural process

This is also known as the action phase where the communal team takes appropriate measures to ensure that cultural rituals are employed correctly.

Assessment carried out in the first phase should determine the process during the current phase. This has to be done with caution because, if it is wrong, it might exacerbate the existing psychosocial challenge.

The elders (‘who’) are normally tasked to head such proceedings. However, they can also be carried out by the father and/or uncles.

#### Psychosocial process

This is where the evaluation takes place. Normally, this happens at a family level where a communal caregiver is assigned to ensure compliance from the communal counsellee; for example, the counsellee can be instructed to perform ‘*Mpho ya Badimo*’ – (‘what’). The caregiver’s role in this regard is to evaluate the initiation plan by the counsellee. The caregiver reports back to the team on the progress depending on the timeline given. Ultimately, the psychosocial process is based on ‘the wheel of life’ being re-aligned again.

In the previous process, specific techniques are used to facilitate healing.

**Techniques –** ‘what and how to say it’.

Notably, these techniques are intertwined in the approach, ‘they hunt together’.

### Communication

What is being said to the counsellee is of importance because of the people constituting the communal team. This team is built depending on the nature of the problem. One of the members should at least be an elder with vast life experience. Covert language is used in the form of metaphors together with stories and thus requires extensive knowledge of life, which brings about the meaning of life. The counsellors should also show the character of being firm and decisive as reflected by boundary setting.

### Silent communication

Elders and individuals in different stages of life happen to naturally acquire this technique of de-escalating emotions with a silent presence. This is, most of the time, used to manage emotional challenges; to be quiet and present in support.

### Humility

As the counsellor, one should display a sense of humility and respect throughout, which is embedded in the principle of *Ubuntu*. It is based on ‘your’ psychosocial challenge is ‘our’ problem. It is accepted that counselees are persons who are allowed to make life mistakes.

### Self-resilience disclosure

In the benefit of empathy, the counsellor should show a sense of openness and transparency. The counsellor shares past experiences on similar psychosocial challenges. This concerns societal challenges and how they have managed these challenges as teachable moments.

## Conclusion

Conclusively, the findings from exploring the therapeutic merits of dialogues vindicate why this KhoiSan community manages to cope with psychosocial challenges even without access to western mental health services. Clearly, therapeutic merits in dialogues of healing as a means of healing in the KhoiSan community do not exist in compartments, but as a ‘gestalt’. It became clear that the benefits of therapeutic merits of dialogues are found in the KhoiSan communality, where togetherness and the sense of humility flourish and sustain the community and thus are part of their everyday life. This forms an important ontological part of the communication system and the balance of life through the integral notion of – ‘what’, ‘why’, ‘who’, ‘whom’, ‘how’ and ‘when’. The emerged conceptual framework will add value in the therapeutic practices, teaching and learning and further research.

## Recommendations

For the revitalisation of the healing practices for the management of psychosocial challenges (objective three), the following recommendations are stated below:

### Communal therapeutic practice

Indigenous African approaches should be integrated into modes of counselling within the clinical setting. Practitioners are expected to augment the skills identified in this approach in appreciating and understanding the techniques of the elders in an African indigenous context, enhancing cultural awareness and sensitivity in mental healthcare.

### Teaching and learning

The researcher recommends that a course be developed and facilitated with a specific focus on re-appropriating indigenous health systems equally into western healthcare systems.

### Further research

Further research is suggested on the advanced development of this approach and its contribution in the revitalisation and re-inscription of such indigenous health practices.

## Limitations

More women participated in the study. However, the KhoiSan community adheres to a ‘motherly’ cultural cosmos of sharing and caring. Additionally, time was a limitation.
